# Emerging *Vibrio vulnificus*-Associated Infections After Seawater Exposure—Cases from the Bulgarian Black Sea Coast

**DOI:** 10.3390/medicina60111748

**Published:** 2024-10-24

**Authors:** Stephanie Radeva, Stoyan Vergiev, Georgi Georgiev, Denis Niyazi

**Affiliations:** 1 Microbiology Laboratory, Multidisciplinary Hospital for Active Treatment “Heart and Brain”, 8000 Burgas, Bulgaria; 2 Department of Microbiology and Virology, Medical University of Varna, 9002 Varna, Bulgaria; denis.niyazi@gmail.com; 3 Department of Ecology and Environmental Protection, Technical University of Varna, 9010 Varna, Bulgaria; stvergiev@gmail.com; 4 Anesthesiology and Intensive Care Ward, Multidisciplinary Hospital for Active Treatment “Heart and Brain”, 8000 Burgas, Bulgaria; georgi522@gmail.com; 5 Microbiology Laboratory, University Multidisciplinary Hospital for Active Treatment “St. Marina”, 9010 Varna, Bulgaria

**Keywords:** *Vibrio vulnificus*, wound infection, septicemia, seawater

## Abstract

*Objectives*: The aim of the current report is to present three cases of necrotizing fasciitis and sepsis caused by *Vibrio vulnificus* on the Bulgarian Black Sea coast. *Materials and Methods*: Two of the patients are males, 70 and 86 years of age, respectively, and one is an 86-year-old female. Data were collected from the patients’ examination records. *V. vulnificus* was isolated on 5% sheep blood agar from wound and blood samples and identified by the automated system Phoenix M50 (BD, Franklin Lakes, NJ, USA). Antimicrobial susceptibility was tested with two well-known methods (disk diffusion and broth microdilution). *Results*: All of the patients were admitted to our hospital due to pain, swelling, ulceration, and bullae on the legs and were febrile. They underwent surgery and received intensive care support. One of the patients developed septicemia and septic shock; one of his legs was amputated, but the outcome was fatal. The other patient received immediate approptiate antibiotic and surgical treatment, and the outcome was favorable. The third patient underwent emergency fasciotomy but died a few hours after admission. *Conclusions*: Global climate change is affecting the distribution of *Vibrio* spp., and their incidence is expected to increase. It is important to highlight the need for awareness among immunocompromised and elderly patients of the potential threat posed by *V. vulnificus* infections.

## 1. Introduction

*Vibrio* species belong to the genus *Vibrio* of the *Vibrionaceae* family. More than 140 species are described, but only a few are known to be involved in human diseases (*V. cholerae*, *V*. *parahaemolyticus*, and *V. vulnificus*) [1,2]. All of them are Gram-negative aquatic micro-organisms, inhabiting warm waters with variable salinity (fresh, brackish, and marine waters). Once in an environment with suitable conditions, they reproduce rapidly, and their numbers increase significantly [3].

Environmental factors that affect *Vibrio* spp. are water temperature and salinity. Almost all members of the genus are halophilic, requiring a certain concentration of salts in the water (NaCl 1–12%) [1]. They prefer alkaline waters (pH 8–9) and survive in wide temperature ranges, from 5 to >40 °C [1,4].

*Vibrio* spp. have global distribution, but their isolation often faces obstacles. One of these is attributed to their ability to form biofilms. When there are insufficient amounts of food sources in the environment, free-living bacteria can attach to various surfaces or other marine organisms and form a polysaccharide slime coat to protect themselves and hydrolase the chitin layer of some copepods [5,6]. In addition, under unfavorable conditions related to temperature (below 18 °C) and water salinity (lower or higher than the optimum), *Vibrio* spp. may undergo transformation and become dormant. In this form, the bacteria are still alive but are not able to form colonies on growth media. These bacterial cells are known as viable but nonculturable (VBNC) [7]. These two factors (biofilm formation and a VBNC state) certainly have an impact on isolating these micro-organisms and determining their incidence in waters in different parts of the world. Thus, non-cultural surveillance methods are recommended [8].

One of the representatives of the family, *V. vulnificus*, is associated with acute, even fatal infections [9]. It is classified into three biotypes (BTs): BT1 is associated with all forms of human infections, BT2 is mainly recovered from eels, and BT3 causes soft tissue infections with a low mortality rate [10].

*V. vulnificus* has two mechanisms of infecting people: the consumption of raw contaminated seafood or the exposure of wounds to seawater [11]. It is believed that foodborne infection caused by *V. vulnificus* is the most common cause of food-associated death. In the United States, its incidence is as high as 95%, surpassing other foodborne diseases such as botulism [12].

According to its clinical presentation, a *V. vulnificus*-associated infection can manifest as wound infection, gastroenteritis, and septicemia. Symptoms of primary sepsis after contaminated seafood consumption include fever, chills, low blood pressure leading to septic shock, and distinctive skin changes like cellulitis, ecchymosis (bruising), and hemorrhagic bullae (blood-filled blisters). The incubation period of this form is about 26 h, and the mortality often exceeds 50% [13].

Wound infections, on the other hand, usually occur after handling contaminated seafood or when open wounds are exposed to contaminated water. Similar to primary septicemia, wound infections can rapidly worsen, leading to cellulitis, ecchymosis, and bullae, potentially developing into necrotizing fasciitis and secondary sepsis. The incubation period of the wound infection is notably shorter (about 16 h), with a mortality rate of 17% [13]. The infectious dose is not known, but presumably less than 100 bacteria are needed to cause disease [14].

Early diagnosis, based on clinical symptoms, diagnostic tests, and the presentation of the infection, along with prompt empiric treatment, are crucial to patient survival. The preferred antimicrobial therapy is doxycycline in combination with third-generation cephalosporin and surgery for necrotizing soft tissue infection [15,16].

## 2. Case Presentation

Herein, we present three cases of *V. vulnificus*-associated wound infections in patients during August and September 2024. All three patients were residents of Bulgarian towns located on the south cost of the Black Sea, had confirmed or suspected exposure to seawater, and had chronic underlying diseases.

Patient A was a 70-year-old man admitted to our hospital due to pain, swelling, ulceration, and bullae on the lower leg, fever up to 40 °C, and lethargy. According to information from his relatives, he had wounds on his leg and was given seawater to soak it in, in order to speed up the healing process. Afterward, his leg began to swell and turned red, and his overall condition worsened, which prompted him to seek medical attention. Upon admission, the patient had tachypnea (>44/min), low blood pressure (80/40 mmHg), an elevated heart rate (100–112/min), and low oxygen saturation (93%); his C-reactive protein (CRP) level was 39 mg/dL, his white blood cell count (WBC) 4.9 × 10^9^/L, and his neutrophile count was 9 × 10^9^/L. A sample from the ulcer, as well as blood cultures, was sent to the microbiology laboratory.

Blood samples were inoculated in special liquid media for cultivation in aerobic (BD BACTEC Plus Aerobic) and anaerobic (BD BACTEC Plus Anaerobic) conditions and incubated in BACTEC FX40 (BD, Franklin Lakes, NJ, USA). When flagged positive, the blood sample was subcultured on 5% sheep blood agar (BD, Franklin Lakes, NJ, USA) and MacConkey agar (BD, Franklin Lakes, NJ, USA) and incubated for 24 h at 37 °C in an environment with 5% CO_2_.

The wound samples were inoculated on blood agar and MacConkey agar under the same conditions. *V. vulnificus* was identified using the NMIC/ID-76 panel for Gram-negative bacteria of the automated system Phoenix M50 (BD, Franklin Lakes, NJ, USA). The Kirby–Bauer disk diffusion method on Mueller–Hinton agar was used to test the susceptibility profile of the isolated micro-organism. A commercial microdilution kit (MIC NEFERM, MIKROLATEST, Erba Lachema, Brno, Czech Republic) was utilized to detect the minimum inhibitory concentration of antibiotics against the *V. vulnificus* isolates. All tests were carried out according to the manufacturer’s instructions, and the results were interpreted according to The European Committee on Antimicrobial Susceptibility Testing (v. 14, 2024) and Clinical and Laboratory Standards Institute (M45 ed. 3, 2018) guidelines. The susceptibility pattern of the isolates is shown in Table 1.

**Figure 1:**
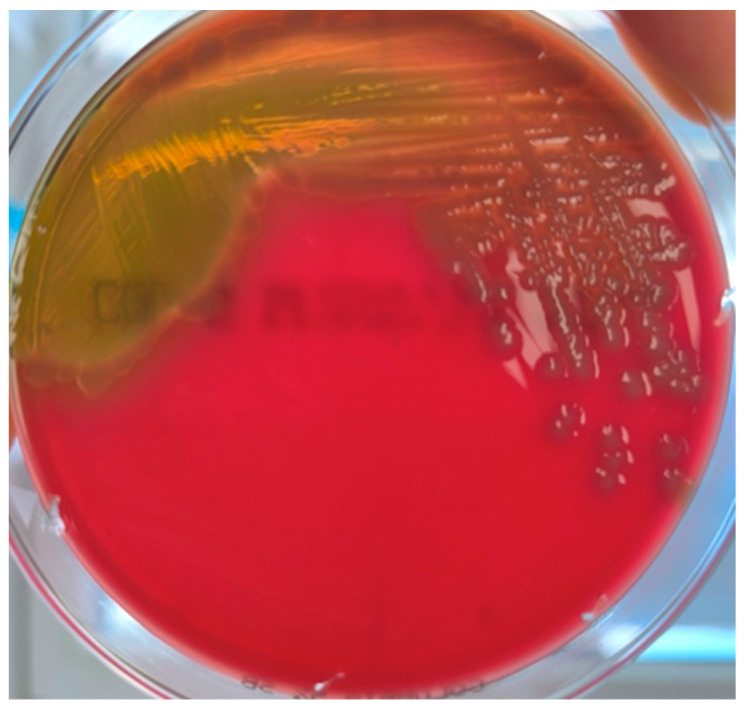
Growth of *V. vulnificus* on 5% sheep blood agar.

The results from the clinical laboratory on the next day showed CRP of 248 mg/dL, WBC of 2.8 × 10^9^/L, and procalcitonin of 32.8 ng/mL. On the third day, CRP escalated to 372 mg/dL, and the patient presented with leukopenia (WBC 2.13 × 10^9^/L) and showed all the signs of septicemia and septic shock. *V. vulnificus* was isolated from both blood and wound cultures. The colonies were round and slightly convex, moist, and gray with green β-hemolysis around them (Figure 1).

**Figure 2:**
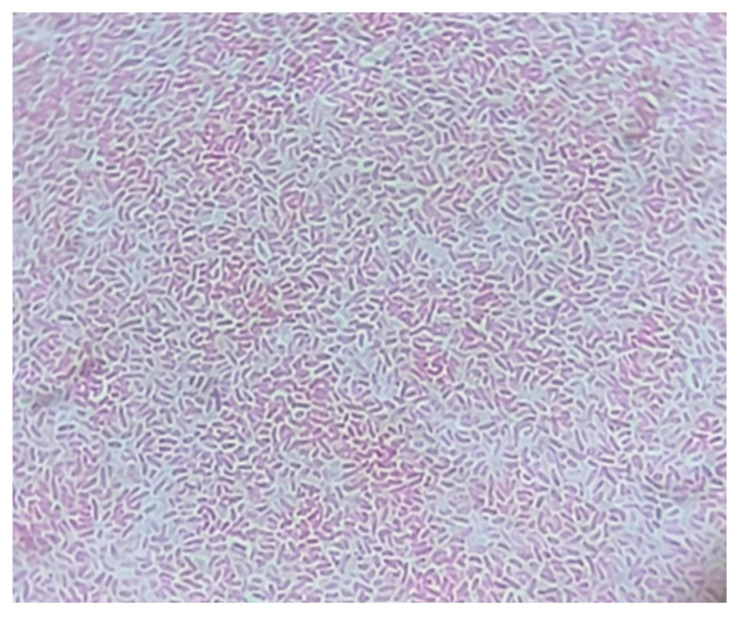
Brightfield microscopy of *V. vulnificus* (Gram-staining, 1000×).

Under the microscope, *V. vulnificus* appeared as a comma-like Gram-negative bacterium (Figure 2).

**Figure 3:**
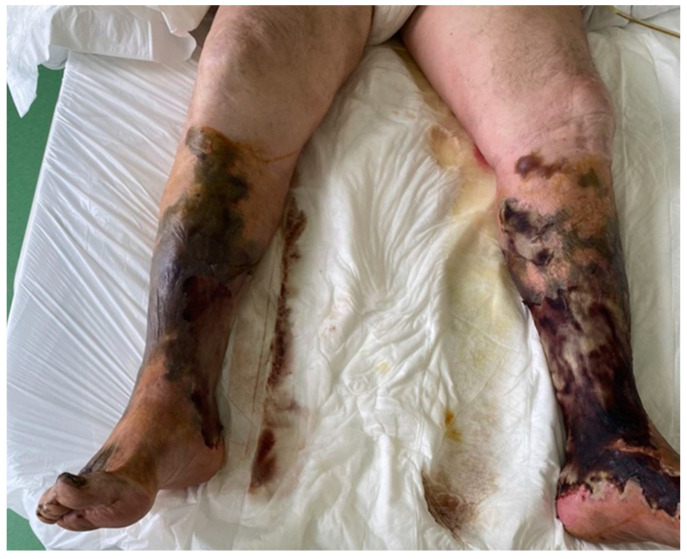
Necrotic lesions in patient A.

The patient was admitted to the emergency intensive care unit (EICU) for further treatment for the septic shock and was intubated. He developed multiple organ dysfunction syndrome (MODS). The other leg presented with the same necrotic changes with bullae (Figure 3).

The patient was treated with meropenem, colistin, doxycycline and levofloxacin, norepinephrine and vasopressin for maintaining blood pressure, rehydration, nadroparin calcium, and antipyretics. The fever was present despite antipiretic and antibiotic therapy. The patient’s left leg was amputated, but the patient’s condition remained critical, and he died a month after admission.

**Figure 4:**
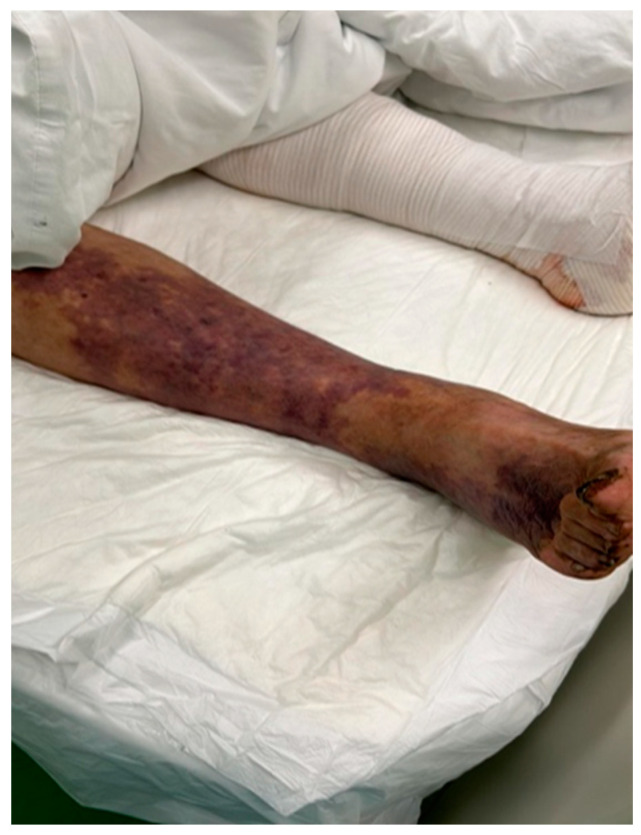
Necrotic bullous changes in the lower leg of patient B.

Patient B was an 86-year-old man admitted to our hospital due to the same symptoms—pain, swelling, and bullae on the legs. From the patient’s history, we could see that he had diabetes, alcoholism, and chronic heart failure and had wounds on his legs due to vascular insufficiency (Figure 4).

He was a fisherman, and after exposure to seawater, one of the legs began to swell and turned red. Because the clinical presentation was the same as that in patient A, *V. vulnificus*-associated infection was suspected by the physicians from the Vascular Surgery Department, which was later confirmed by the microbiology laboratory. The isolate was cultured and identified using the same methods as already described and demonstrated the same susceptibility pattern as the first isolate. Antibiotic treatment with doxycycline and meropenem was started immediately, in combination with nadroparin calcium, pantoprazole, and furosemide. The results from the clinical laboratory at admission were as follows: CRP, 178 mg/dL (elevating to 329 mg/dL on the next day); WBC, 13.5 × 10^9^/L; and neutrophils, 12.1 × 10^9^/L. He had tachypnea −25/min, a blood pressure of 95/60 mmHg, oxygen saturation at 95%, and a heart rate of 115/min. Surgery was performed on the affected area, after which the wound was cared for daily. On the seventh day, the patient was hemodynamically stable, but the treatment continued. On the 10th day, the CRP dropped to 10.1 mg/dL. Despite the skin excision, the wound did not heal, nor were there any signs of granulation tissue, and the necrosis continued to expand, which led to the amputation of one of the legs. The patient was in hospital until he reached full recovery and was discharged one month after admission.

**Figure 5:**
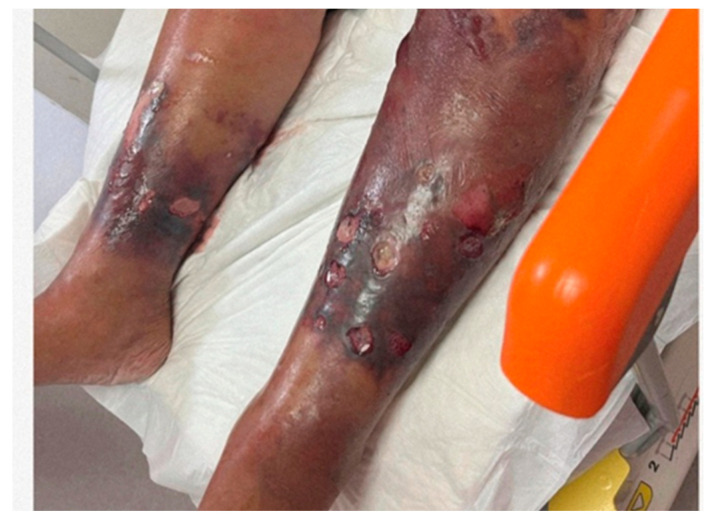
Lower leg changes in patient C.

Patient C was an 86-year-old woman admitted due to pain, swelling, ulcers, and bullae on both of her legs (Figure 5). She had diabetes, chronic cardiovascular disease, and chronic wounds on the legs and was disorientated on admission. According to her relatives’ information, her legs began to swell, and bullae appeared 24 h before admission. Seawater exposure was suspected, but the information given by relatives was lacking and unclear. Upon admission, the patient had tachypnea (>35/min), a blood pressure of 60/30 mmHg, a heart rate of 140/min, oxygen saturation of 92%, a CRP level of 330.1 mg/dL, a WBC count of 7.92 × 10^9^/L, and a neutrophil count of 7.3 × 10^9^/L. Samples from the ulcers were sent to the microbiology laboratory. The patient showed all the signs of shock. Immediate treatment with doxycycline, norepinephrine and vasopressin, rehydration, and nodraparine calcium was started. Despite the emergency fasciotomy, she died hours after admission. From the wound secretions, *Proteus vulgaris*, *Klebsiella pneumoniae*, and *V*. *vulnificus* were isolated.

The comparative characteristics of the three patients are presented in Table 2.

## 3. Discussion

*Vibrio vulnificus* is an opportunistic pathogen causing severe life-threatening infections following the consumption of seafood or the exposure of wounds to *Vibrio*-infested waters [1].

*Vibrio vulnificus* infections occur with the highest incidence in summer, when sea surface temperatures range from 23 to 29 °C [16,17]. Only a few isolated cases of infections after exposure to colder sea water have been described in the literature [18,19].

Bulgaria is in the temperate continental climate zone [20]. The summer spans from late June to early September, when the temperature of the coastal waters of the Black Sea reaches 24–26 °C, and this is the period in which the cases described in the current report occurred [21,22].

In a two-year German study investigating the temporal and annual distribution of potentially pathogenic *Vibrio* spp. in coastal waters, a higher frequency of the bacteria was reported during the summer months, with *V. vulnificus* only recovered at water temperatures between 14 and 26.5 °C [23]. In another European study, *V. vulnificus* was detected in July-September, with higher incidence in coastal waters compared to open waters [24].

In a study on coastal waters of the Black Sea and some freshwater reservoirs, there was evidence of a higher frequency of *Vibrio* spp. in seawater, with *V. vulnificus* detected in 4% of the samples [25]. These studies confirm the influence of water temperature and salinity on the activity of *V. vulnificus* in the regions where our cases occurred. In contrast to temperate climates, such a dependence has not been demonstrated in the equatorial and tropical zones, where *V. vulnificus* is detected all year round. In these regions, salinity is accepted as the more significant environmental factor [1].

As a typic halophile, *V. vulnificus* requires water salinity between 8 and 16 ppt, and a NaCl concentration between 0.5 and 5% [1]. With a salinity of 16–17 ppt, a NaCl content of around 3% and a temperature reaching 24–26 °C during the summer months in recent years, the Black Sea waters along the Bulgarian coast are an ideal environment for *V. vulnificus* to thrive in [22].

It has been reported that *V. vulnificus* enters into associations with the plankton found in marine waters [26]. During the bloom of microalgae, a large amount of nutrients are released for the *Vibrio* spp. to use, and the clustering of the plankton together allows the bacteria to use them as attaching sites [26]. Starting in May, before the three cases occurred, a mass phytoplankton bloom was reported in the coastal waters of the Black Sea [27].

All three of our patients had underlying chronic diseases and wounds on the lower extremities. It is known that there are certain risk factors that predispose individuals towards *V*. *vulnificus*-associated infection and its severe course, such as immunosuppression, liver cirrhosis, alcoholism without cirrhosis, diabetes, and advanced age [28]. All our patients were elderly (>65 years of age), two of them had diabetes, and one reported alcohol abuse.

According to the patients or their relatives, the symptoms appeared after exposure to seawater and rapidly worsened, which is in concordance with the *V. vulnificus* infections and may be due to both the chronic diseases of the individuals and the virulence of the pathogen.

As a Gram-negative bacterium, *V. vulnificus* contains lipopolysaccharides (LPSs) in its outer membrane. These structures are known to play the role of endotoxin and are associated with hypotension and septic shock. It has been confirmed in animal models that the inhibition of the effect of LPSs leads to the survival of experimental animals [13].

Multiple virulence factors have been shown to confer attachment and invasiveness on the bacterium or its ability to evade the host immune system [1,13,28]. Some strains produce a polysaccharide capsule with antiphagocytic activity [13]. Only encapsulated strains have been shown to be pathogenic, since the capsule is involved in adhesion to the host’s cells [29]. Also, once in the bloodstream, the capsule-coated *V. vulnificus* resists the bactericidal properties of the blood [10]. The micro-organism can actively secrete an exotoxin outside the cell [30]. It is known as *V. vulnificus* hemolysin (VVH) and belongs to the cholesterol-dependent cytolysins [31]. Its role in infection is related to cytotoxicity and pore formation in eukaryotic cells (e.g., red blood cells); it has an effect on osmotic pressure and leads to vasodilation with subsequent hypovolemic septic shock [32]. In addition, the production of enzymes such as collagenase, metalloprotease, or phospholipase has been demonstrated to be associated with the invasive capability of the bacterium [10].

Despite timely antibiotic therapy and surgical intervention, two of the three patients did not survive. Mortality has been reported to increase with the delay in antibiotic therapy. When the etiologic treatment is initiated between 48 and 72 h and >72 h after admission, the mortality rate is reported to reach 63% and 100%, respectively. If antibiotic treatment is started at the time of admission, mortality can decrease to 33% [33]. In patients with soft tissue involvement, surgical debridement is also needed. In a study involving patients with necrotizing fasciitis caused by *V. vulnificus*, the group undergoing surgery within 12–24 h after admission had a better survival rate than the group with a delay of >24 h [34]. We suspect that the main factor that influenced the unfavorable outcome in our two cases was the delay in seeking medical attention.

In our study, the antibiotic susceptibility of the three isolates was preserved, which was confirmed by two methods. Resistance was demonstrated only to colistin, which is consistent with other reports [9].

Various cases of *V. vulnificus* infection have been described in Europe over the years, in France [35], Germany [36], Spain [37], and Italy [38]. According to the European Food Safety Authority’s (EFSA) latest assessment, the prevalence of *Vibrio* in seafood is expected to increase both globally and in Europe because of climate change, especially in low-salinity or brackish waters [39]. Over the last two decades, Europe has experienced an increase in *Vibrio* infections [40]. Rising temperatures in coastal waters have expanded the regions where *Vibrio* bacteria thrive, raising the likelihood of infections from eating contaminated seafood [40]. The areas most vulnerable to this risk include those with brackish or low-salinity waters, such as the Baltic Sea, the transitional waters of the Baltic and North Seas, and the Black Sea, as well as coastal zones with significant river inflows [25,41,42].

After a thorough literature review, a single Bulgarian case, reported in 2020, concerning a Belgian tourist who visited the south coastal region of the Black Sea, was documented [43]. Similar to our cases, the patient had underlying chronic disease (type 1diabetes) and, despite medical attention, succumbed to the *V. vulnificus* infection.

The lack of described cases in Bulgaria in previous years may be due to the lower average annual temperature (below 13 °C) of the surface coastal waters [22]. The change in climate conditions leading to the warming of the waters and the retention of higher temperatures for a longer period of time may become a factor leading to more frequent encounters with this pathogen and the occurrence of new cases during the summer months in Bulgaria.

As far as we know, this is the second reported case of *Vibrio vulnificus* from our country.

## 4. Conclusions

Our experience shows the crucial role of immediate adequate antibiotic treatment, as well as proper surgical intervention, for increasing the chances of survival for patients with *V. vulnificus*-associated infection. Due to global climate change, the prevalence of *Vibrio* spp. is expected to increase, both globally and in Europe, so this issue is of great public significance. This is why the population needs to be informed about prevention methods. Immunocompromised individuals, such as patients with chronic diseases or cancer, those who have recently undergone transplantation, those with diabetes, or elderly patients, should wear proper foot protection to prevent cuts and injury caused by rocks and shells on the beach. Furthermore, they should avoid seawater exposure if any wounds are already present, as well as the consumption of raw or undercooked seafood.

## Figures and Tables

**Table 1:** Susceptibility pattern of the three *V. vulnificus* isolates.

	Antibacterial Agents																
	AMS	PIP	PIT	CTX	CAZ	FEP	AZT	IPM	MER	GEN	AK	CL	CIP	LVX	DOX	SXT	TIG
MIC (µg/mL)	2/1	<1	<1	0.25	0.5	N	2	0.5	0.25	0.5	1	>16	<0.06	<0.06	N	<0.06/1.1	<0.06
Zone (mm)	20	N	27	30	23	25	N	28	30	20	21	6	26	25	23	25	N
EUCAST	nd	nd	S	S	S	nd	nd	nd	S	nd	nd	nd	S	S	S	S	nd
CLSI	S	S	S	S	S	S	nd	S	S	S	S	nd	S	S	nd	S	nd

AMS—ampicillin/sulbactam; PIP—piperacillin; PIT—piperacillin/tazobactam; CTX—cefotaxime; CAZ—ceftazidime; FEP—cefepime; AZT—aztreonam; IPM—imipenem; MER—meropenem; GEN—gentamicin; AK—amikacin; CL—colistin; CIP—ciprofloxacin; LVX—levofloxacin; DOX—doxycycline; SXT—sulfamethoxazole/trimethoprim; TIG—tigecycline; S—susceptible; N—not tested; nd—not determined by guidelines.

**Table 2:** Main characteristics of the three patients.

	Patient A	Patient B	Patient C
Sex	Male	Male	Female
Age	70	86	86
Anamnesis for water exposure	Yes	Yes	Suspected
Comorbidities	Vascular insufficiency, wounds	Diabetes, alcoholism, chronic heart failure, vascular insufficiency, wounds	Diabetes, vascular insufficiency, wounds
Clinical laboratory results at admission	CRP 39 mg/dL, WBC 4.9 × 10^9^/L, NEU 9 × 10^9^/L	CRP 178 mg/dL, WBC 13.5 × 10^9^/L, NEU 12.1 × 10^9^/L	CRP 330.1 mg/dL, WBC 7.92 × 10^9^/L, NEU 7.3 × 10^9^/L
Heart rate	100–112/min	115/min	140/min
Blood pressure	80/40 mmHg	95/60 mmHg	60/30 mmHg
O_2_ saturation	93%	95%	92%
Respiratory rate	44/min	25/min	>35/min
Treatment outcome	Fatal	Favorable	Fatal

CRP, C-reactive protein; WBC, white blood cell count; NEU, neutrophil count.

## Data Availability

All data are presented in the manuscript.
